# Audiological Manifestations in Patients with Granulomatosis with Polyangiitis

**DOI:** 10.3390/medicina60020267

**Published:** 2024-02-03

**Authors:** Vija Vainutienė, Justinas Ivaška, Jolanta Dadonienė, Vilma Beleškienė, Tatjana Ivaškienė, Eugenijus Lesinskas

**Affiliations:** 1 State Research Institute Centre for Innovative Medicine, Santariškių str. 5, LT-08406 Vilnius, Lithuania; jolanta.dadoniene@mf.vu.lt (J.D.); tatjana.ivaskiene@imcentras.lt (T.I.); 2 Clinic of Ear, Nose, Throat and Eye Diseases, Institute of Clinical Medicine, Faculty of Medicine, Vilnius University, M.K. Čiurlionio str. 21, LT-03101 Vilnius, Lithuania; justinas.ivaska@santa.lt (J.I.); vilma.beleskiene@santa.lt (V.B.); eugenijus.lesinskas@santa.lt (E.L.); 3 Department of Public Health, Faculty of Medicine, Vilnius University, M.K. Čiurlionio str. 21, LT-03101 Vilnius, Lithuania

**Keywords:** vasculitis, granulomatosis with polyangiitis, hearing loss, audiometry

## Abstract

*Background and Objectives*: Granulomatosis with Polyangiitis (GPA) is a rare, autoimmune, multisystemic disease characterized by vasculitis and necrotizing granuloma that commonly affects the upper and lower respiratory tract and kidneys. Audiovestibular dysfunction in GPA diseases may have different clinical presentations. The aim of the present study was to evaluate hearing function in patients with GPA and to compare the results with a healthy control group. *Materials and Methods*: A total of 34 individuals participated in the study. The GPA group consisted of 14 participants, and the control group was composed of 20 healthy participants with no signs or symptoms of ear disease. The ages ranged from 18 to 65 years old, with a mean age of 43.8 years. The participants underwent a complete audiological evaluation using otoscopy, impedance audiometry, pure tone audiometry, speech audiometry—evaluation of speech thresholds, and speech recognition in quiet. Both ears were tested. All of the participants of the study were native Lithuanian speakers. Data were statistically analyzed using the Statistical Analysis System software SAS^®^ Studio 3.8. A *p* value < 0.05 was regarded as statistically significant. *Results*: 92.85% of patients from the GPA group reported hearing-related symptoms: hearing loss, tinnitus, and fullness in the ears. The arithmetic means of all hearing thresholds at frequencies from 125 Hz to 8000 Hz were significantly higher in the GPA group. The results revealed statistically significant differences between the two groups in the Speech Detection Threshold, Speech Recognition Threshold, Speech Discomfort level, and Word Recognition Scores. *Conclusions*: The frequency of hearing loss, the average hearing thresholds, and speech thresholds were higher in GPA patients than in healthy individuals. The most common type of hearing loss was sensorineural. Audiological assessments should be considered during the routine evaluation of patients with GPA disease to prevent hearing-related disabilities.

## 1. Introduction

Granulomatosis with polyangiitis (GPA), also known as Wegener‘s granulomatosis, is a rare, autoimmune, multisystemic disease characterized by vasculitis and necrotizing granuloma, mostly affecting the upper and lower respiratory tract and kidneys [1]. The disease was first reported by Heinz Klinger in 1931 and later described by Friedrich Wegener in 1936 [2,3]. GPA is a small vessel vasculitis and belongs to anti-neutrophil cytoplasmic antibody (ANCA)-associated vasculitis [4]. The pathogenesis of GPA is complex, with important roles for ANCA, cellular immunity, neutrophils, fibroblasts, vascular endothelial cells, and inflammatory mediators [5,6].

The prevalence of GPA is 3–14 cases per million population per year, and the disease can occur in all racial groups, but Caucasians are the most affected [7,8]. The disease can appear at any age, and it affects both genders [9].

Patients with GPA usually report fatigue, night sweats, weight loss, and respiratory symptoms such as cough, recurrent sinusitis, epistaxis, and hemoptysis. Otorhinolaryngological symptoms may be the first clinical manifestation of the disease, as the upper respiratory tract is often affected [10,11]. The nasal cavity and the paranasal sinuses are the most frequent sites of GPA in the head and neck region [12]. Otologic manifestations occur in 8–65% of patients with GPA [13,14,15]. Otologic involvement in GPA can range from serous otitis media to sensorineural hearing loss and can occur during the course of the disease, in addition to being the first and only sign of GPA [16,17].

Hearing loss in GPA can vary in severity and can affect one or both ears. Delayed diagnosis and therapy can negatively affect the prognosis of hearing. Early detection and appropriate medical management are important to control inflammation and minimize the impact on hearing. In the medical literature, the analysis of the relationship between GPA and hearing loss is mostly based on pure tone audiometry. Data on the level of speech perception in patients with GPA, as determined by speech audiometry, are scarce, and no such study has been carried out in Lithuania so far. 

The aim of the present study was to investigate audiological manifestations and to study the hearing status of patients with GPA.

## 2. Materials and Methods

A total of 34 individuals participated in the study. The GPA group consisted of 14 participants, and the control group consisted of 20 healthy participants with no signs or symptoms of ear disease. Both ears were tested. All of the participants of the study were native Lithuanian speakers. The diagnosis of GPA was confirmed at Vilnius University Santaros Clinics, Department of Rheumatology. At the time of diagnosis, the disease was classified as GPA if it met the 1990 ACR classification criteria [18]. Patients diagnosed with GPA between 2007 and 2023 were included in the study. Before inclusion into the study, it was ensured that all the patients met the 2022 ACR/EULAR classification criteria for GPA [19]. ANCA was detected using an indirect immunofluorescence assay and, if positive, was confirmed via an antigen-specific enzyme-linked immunosorbent assay. All 14 patients with GPA underwent biopsies (sometimes multiple biopsies of different organs), and in 12 cases, a biopsy revealed granulomatous inflammation, consistent with the diagnosis of GPA or pauci-immune glomerulonephritis in the case of renal biopsy. In 2 cases, the biopsy results were non-specific, and the diagnosis of GPA was made on the basis of other criteria, such as a typical clinical presentation and an ANCA-positive result. The hearing assessment was carried out at Vilnius University Hospital Santaros Clinics, Department of Ear, nose and throat diseases between December 2022 and December 2023. All audiograms were reviewed and interpreted by the same otorhinolaryngologist.

The participants underwent a comprehensive audiological assessment comprising otoscopy, impedance audiometry, pure tone audiometry, and speech audiometry. The examination of the ear canal and the tympanic membrane was performed using an otoscope. The external ear, ear canal, and tympanic membrane were examined for any abnormalities or signs of infection. Tympanometry was performed using a Homoth impedance audiometer at a probe tone frequency of 226 Hz. The compliance of the tympanic membrane and the pressure in the middle ear were measured to identify conditions such as otitis media or Eustachian tube dysfunction. The results of tympanometry were presented in a graph showing compliance, gradient, and pressure. According to the normative values, the tympanograms were classified as Type A, Type B, or Type C curves [20]. Type A is a bell-shaped curve with maximum compliance at +50 to −100 daPa and indicates normal middle ear pressure. The Type B tympanogram is described as a flat curve with no maximum point of compliance. The most common cause of this pattern is reduced mobility of the tympanic membrane due to fluid in the middle ear. The type B tympanogram also corresponds to other pathology of the middle ear, such as tympanosclerosis, cholesteatoma, and middle ear tumors. The type C tympanogram is characterized by a bell-shaped curve in the negative pressure range (below −100 daPa) and indicates impaired Eustachian tube function [21].

Audiological examinations were performed in a double-walled sound-treated booth using a calibrated clinical audiometer (Interacoustics AC40, Assens, Denmark). Pure tone audiometry is a basic test to assess a person’s hearing thresholds at different frequencies. It provides pure tones of different frequencies and intensities to identify the softest sounds a person can hear. Pure tone audiometry was performed at frequencies of 125, 250, 500, 1000, 2000, 4000, and 8000 Hz both using air and bone conduction. The softest sound a person can hear at a given frequency is called a hearing threshold. In order to assess hearing sensitivity and to provide diagnostic information on the severity, type, and configuration of hearing loss, pure tone thresholds in air and bone were determined. Based on the results of pure tone audiometry, hearing loss was classified into conductive, sensorineural, and mixed. Conductive hearing loss (CHL) was defined when the air-bone gap was greater than 10 dB. Sensorineural hearing loss (SNHL) was defined when both the air and bone conduction thresholds were less than 15 dB with an air–bone gap less than 10 dB. Mixed hearing loss (MHL) was defined as a combination of conductive and sensorineural components [22].

Speech audiometry is used to assess participants’ speech perception abilities. It helps to determine the individual’s Speech Detection Threshold (SDT), Speech Recognition Threshold (SRT), Word Recognition Score (WRS), and Speech Discomfort Level (SDL). SDT is the lowest level at which the speech signal is heard. SRT is the intensity at which the patient understands and correctly repeats 50% of the items on the list. WRS is the percentage of test words that the patient repeats correctly. The WRS is administered at a comfortable listening level and assesses the person’s ability to recognize and understand spoken words. SDL is the level at which the speech sounds become uncomfortably loud or when the person feels discomfort [23]. Speech audiometry tests were administered using speech audiometry materials in the Lithuanian language. The speech stimuli were routed from a CD player (Panasonic DVD-S42, Dalian, China) to the clinical audiometer and delivered to the participants via TDH−39 headphones. For speech recognition, each participant was presented with a list of 25 bisyllabic phonetically balanced words in quiet. WRS was calculated by the percentage of correctly repeated words. SDL was measured by asking participants to indicate their discomfort in response to increasing levels of speech stimuli.

Data were statistically analyzed using the Statistical Analysis System software SAS^®^ Studio 3.8 (Copyright © SAS Institute Inc., Cary, NC, USA). Descriptive statistics for continuous variables were presented as a mean ± standard deviation (SD). Differences between the groups were examined using two-way repeated measures ANOVA with the ear as within-subject and the group as between-subject factors. A *p* value < 0.05 was considered statistically significant. 

The study was approved by the Vilnius Regional Biomedical Research Ethics Committee of Lithuania. The participants signed an informed consent form at the beginning of the study.

## 3. Results

Thirty-four individuals (17 females and 17 males) took part in the study. The ages ranged from 18 to 65 years old, with a mean age of 43.8 years. A detailed description of the participants is given in Table 1. 

The mean duration of GPA was 2.8 years, ranging from 0.5 to 15 years. Overall, 71.4 % of the GPA cases were positive for ANCA. 

Hearing-related symptoms such as hearing loss, tinnitus, fullness of the ears, and otalgia were reported by 92.9% of the GPA group (Table 2).

71.4% of patients in the GPA group had a type A tympanogram, 17.9% had a flat tympanogram (type B), and 10.7% had a non-measurable acoustic impedance due to the presence of the ventilation tube and the blockage of the external auditory canal by an exostosis. All control group subjects had normal tympanograms. In 60.7% of the GPA patients, the pure tone audiogram showed some degree of hearing loss. The most common type of hearing impairment was sensorineural (32.1%); 21.4% of the GPA patients had mixed hearing loss, and 7.1% had conductive hearing impairment. The arithmetic means of all hearing thresholds at all frequencies from 125 Hz to 8000 Hz were significantly higher in the GPA group compared to the controls (Table 3). 

The highest hearing thresholds were observed in the GPA group with mixed hearing impairment (Table 4).

The speech audiometry test showed that the mean SDT was 32.9 ± 17.2 dB, SRT was 43.6 ± 17.6 dB, WRS was 97.4 ± 5.7%, and SDL was 97.9 ± 8.9 dB in the GPA group. The results in the control group were as follows: SDT 17.5 ± 4.2 dB, SRT 27.4 ± 3.9 dB, WRS 99.5 ± 1.3%, and SDL 101.1 ± 3.7 dB. The results of the GPA and control groups were compared using the analysis of variance model (ANOVA) for repeated measures. The results revealed statistically significant differences between the values of SDT, SRT, SDL, and WRS for both groups (Table 5). 

## 4. Discussion

Otological manifestations, including chronic otitis media and sudden or progressive sensorineural hearing loss, have been described in various autoimmune diseases, such as rheumatoid arthritis, Sjogren’s syndrome, systemic lupus erythematosus, polyarteritis nodosa, Churg–Strauss syndrome, temporal arteritis, Takayasu arteritis, Kawasaki disease, and Cogan‘s syndrome [24,25,26,27,28,29,30,31,32,33]. 

GPA is a rare, autoimmune disease that primarily affects small- to medium-sized blood vessels. The clinical manifestations of GPA are heterogeneous. While it predominantly involves the respiratory tract and kidneys, it can also affect various other organs, including the ears. The otological manifestations of GPA can have significant implications for hearing status. Hearing loss can have a significant impact on a patient’s quality of life, affecting communication, social relationships, and general well-being. According to the literature, otological signs are present in 8–65% of patients with GPA, mainly as auditory symptoms in the form of SNHL or CHL [13,14,34]. CHL in patients with GPA may develop due to granulation tissue affecting the middle ear, nasopharynx, or Eustachian tube [16,35]. Inner ear involvement, which leads to SNHL in GPA, is due to inner ear vasculitis, capillary occlusion, thickened vessel walls, and hemorrhages within the stria vascularis, spiral ligaments, and vestibule [36,37,38]. 

The incidence of audiological involvement in GPA varies across patient populations and studies. In a study by Wali et al., hearing impairment was found in 35% of patients with GPA [13]. Bakthavachalam et al. reported that 56% of patients with GPA had hearing loss, and the most common hearing loss was sensorineural in 47% of cases. The data by Bakthavachalam et al. indicate that other factors, such as age-related changes in hearing, may have influenced the results of the hearing tests performed on GPA patients in their study [39]. Age-related hearing loss, also known as presbycusis, is the most common cause of SNHL in elderly patients [40]. The age of the participants in our study ranged from 18 to 65 years. The patients over 65 years were excluded from our study to avoid age-related hearing loss. Our study revealed that 60.7% of patients with GPA had some degree of hearing loss in the pure tone audiogram. The most common type of hearing loss was sensorineural (32.1%). The highest hearing thresholds were observed in the GPA group with MHL.

Diagnosing the otological manifestations in GPA can be challenging, as symptoms may be non-specific and overlap with other ear disorders [41]. Hearing assessments in individuals with GPA require both pure tone audiometry and speech audiometry. Pure tone audiometry can help to identify any hearing loss associated with GPA disease and determine the degree and configuration of the hearing loss. Speech audiometry helps to assess patients’ ability to hear and understand specific types of speech stimuli [42]. Speech audiometry tests performed in our study indicated that patients with GPA had higher speech thresholds compared to the control group. The results revealed statistically significant differences between the GPA and control groups in SDT, SRT, SDL, and WRS.

The diagnostic challenges discussed by Thornton et al. highlight the importance of a combination of otoscopic examination, audiometric tests, and imaging studies to assess the nature and extent of hearing impairment [43]. Usually, otologic symptoms are non-specific findings, ranging from mild inflammation to profound hearing loss, and may affect one or both ears. A review by Kempf et al. emphasizes the clinical heterogeneity of GPA, including the variable manifestation of hearing loss [44]. Patients may experience a gradual onset of symptoms or a sudden decline in hearing function. Otological symptoms may include tinnitus, dizziness, and fullness in the ears. Ear involvement may occur during the course of the disease, but it should be noted that hearing loss may be the initial symptom of GPA [17,45,46]. The study by Bakthavachalam et al. suggests that GPA should be considered if hearing loss is permanent or acute [39]. 

In our study, 92.9% of patients with GPA reported hearing-related symptoms, such as hearing loss, tinnitus, fullness in the ears, and otalgia. Our study revealed that otological symptoms as initial manifestations of GPA have been observed in five patients (35.7% cases): two patients presented with GPA as acute serous otitis media, two others were diagnosed with GPA after persistent purulent inflammation of the middle ear, and in one case, GPA was diagnosed at the onset of sensorineural hearing loss with facial nerve palsy. A study by Fauci et al. revealed that serous otitis media was the initial symptom of GPA in 25% of GPA patients, and hearing loss was the initial symptom in 6% of cases [47]. 

## 5. Conclusions

Based on our study, we conclude that GPA patients had a higher prevalence of hearing impairment and a higher mean hearing threshold than healthy subjects. The most common type of hearing loss in patients with GPA was sensorineural. The results revealed statistically significant differences between the two groups in SDT, SRT, SDL, and WRS. Hearing assessments of individuals with GPA disease play a key role in determining the impact of the disease on their hearing function. Speech audiometry and pure tone audiometry are valuable tools in this assessment process. The early detection of hearing problems and appropriate management of hearing issues can significantly improve the quality of life of individuals with this rare autoimmune disease; therefore, audiological assessments should be taken into account during the routine assessment of patients with GPA to prevent hearing-related disabilities. 

## Figures and Tables

**Table 1:** Characteristics of study participants.

			GPA Group (N- 14)	Control Group (N- 20)
Age, average ± SD			47.2 ± 10.1	40.4 ± 9.2
Sex	Female		6 (42.9%)	11 (55.0%)
	Male		8 (57.1%)	9 (45.0%)
Disease duration, years (range)			2.8 (0.5–15)	-
ANCA-positive			10 (71.4%)	-
Medication usage (ever)		Glucocorticoids	14 (100%)	-
		Immunosuppressants	13 (92.9%)	
		Biologicals	4 (28.6%)	
Organ involvement (ever)		Ear, nose, throat	14 (100%)	-
		Lung	10 (71.4%)	
		Kidney	6 (42.9%)	
		Nervous system	3 (21.4%)	
		Musculoskeletal system	1 (7.1%)	
		Skin	1 (7.1%)	
		Eye	1 (7.1%)	

**Table 2:** Frequency of otological symptoms in GPA group.

Symptom	Number (%)
Hearing loss	8 (57.1)
Tinnitus	7 (50.0)
Fullness in the ears	6 (42.9)
Otalgia	2 (14.3)

**Table 3:** The arithmetic means (± standard deviation (SD)) of pure tone audiometry hearing thresholds at frequencies from 125 Hz to 8000 Hz in patients with GPA and control groups.

Frequency	GPA Group (dB) N = 14	Control Group (dB) N = 20	
	Mean ± SD	Mean ± SD	*p* Value
125 Hz	18.4 ± 12.3	6.0 ± 3.0	<0.0001
250 Hz	18.9 ± 15.1	6.6 ± 3.5	0.0002
500 Hz	21.3 ± 17.2	7.3 ± 3.2	0.0003
1000 Hz	21.6 ± 16.7	7.9 ± 3.2	0.0002
2000 Hz	23.9 ± 19.9	8.5 ± 4.3	0.0004
4000 Hz	33.8 ± 24.3	10.1 ± 3.8	<0.0001
8000 Hz	35.9 ± 29.9	11.5 ± 3.4	0.0003

**Table 4:** The arithmetic means (±SD) of pure tone audiometry hearing thresholds at frequencies from 125 Hz to 8000 Hz in patients with GPA in different types of hearing impairment.

Frequency	Type of Hearing Impairment			
	Normal Hearing	CHL	SNHL	MHL
	Mean ± SD	Mean ± SD	Mean ± SD	Mean ± SD
125 Hz	11.0 ± 3.9	32.5 ± 3.5	15.6 ± 4.2	26.9 ± 17.7
250 Hz	10.5 ± 3.7	35.0 ± 14.1	13.8 ± 4.4	30.6 ± 21.1
500 Hz	10.0 ± 3.3	37.5 ± 3.5	14.4 ± 3.2	38.1 ± 22.0
1000 Hz	11.0 ± 3.2	37.5 ± 10.6	15.6 ± 9.0	38.9 ± 20.7
2000 Hz	11.0 ± 3.9	40.0 ± 0.0	16.3 ± 9.2	43.1 ± 25.2
4000 Hz	14.0 ± 7.0	35.0 ± 0.0	34.4 ± 17.4	57.5 ± 26.7
8000 Hz	14.5 ± 5.0	47.5 ± 24.7	30.6 ± 20.6	65.0 ± 34.9

**Table 5:** Speech audiometry results in GPA and control groups.

	SDT (dB)	SRT (dB)	WRS (%)	SDL (dB)
	Mean ± SD	Mean ± SD	Mean ± SD	Mean ± SD
**GPA group**	32.9 ± 17.2	43.6 ± 17.6	97.4 ± 5.7	97.9 ± 8.9
**Control group**	17.5 ± 4.2	27.4 ± 3.9	99.5 ± 1.3	101.1 ± 3.7
***p*** **value**	<0.001	<0.001	0.03	0.04

## Data Availability

The dataset collected and/or analyzed in the present study is available upon request from the corresponding author.
